# Probe-Integrated Label-Free Electrochemical Immunosensor Based on Binary Nanocarbon Composites for Detection of CA19-9

**DOI:** 10.3390/molecules27206778

**Published:** 2022-10-11

**Authors:** Zhengzheng Yan, Jun Xing, Ruochong He, Qinping Guo, Ji Li

**Affiliations:** 1 General Surgery Department, Shanxi Bethune Hospital, Shanxi Academy of Medical Sciences, Tongji Shanxi Hospital, Third Hospital of Shanxi Medical University, Taiyuan 030032, China; 2 Department of Breast Surgery, Shanxi Bethune Hospital, Shanxi Academy of Medical Sciences, Tongji Shanxi Hospital, Third Hospital of Shanxi Medical University, Taiyuan 030032, China

**Keywords:** electrochemically reduced graphene oxide, carbon nanotubes, methylene blue, CA19-9, immunosensor

## Abstract

Convenient and sensitive detection of tumor biomarkers is crucial for the early diagnosis and treatment of cancer. Herein, we present a probe-integrated and label-free electrochemical immunosensor based on binary nanocarbon composites and surface-immobilized methylene blue (MB) redox probes for detection of carbohydrate antigen 199 (CA19-9), which is closely associated with gastric malignancies. Nanocarbon composites consisting of electrochemically reduced graphene oxides and carbon nanotubes (ErGO-CNT) are electrodeposited onto an indium tin oxide (ITO) electrode surface to form a 3D nanocomposite film, which could provide high surface area to immobilize abundant MB probes, facilitate the electron transfer of MB, and therefore, improve sensitivity. Polydopamine (PDA) served as a bifunctional linker is able to immobilize anti-CA19-9 antibodies and stabilize the inner probe, conferring the sensing interface with specific recognition capacity. Electrochemical detection of CA19-9 is achieved based on the decrease of the redox signal of MB after specific binding of CA19-9 with a wide linear range of 0.1 mU/mL to 100 U/mL and a limit of detection (LOD) of 0.54 nU/mL (S/N = 3). The constructed electrochemical immunosensor has good selectivity, repeatability, reproducibility, and stability. Furthermore, determination of CA19-9 in human serum samples is also realized.

## 1. Introduction

Ultrasensitive and specific detection of tumor markers is crucial for the early diagnosis and treatment of cancer [1,2]. Generally, abnormal presence and changes of concentrations of tumor markers means that people have suffered from a deadly cancer [3]. Carbohydrate antigen 19-9 (CA19-9) is a kind of carbohydrate antigen consisting of macromolecular glycoproteins and has been shown to be closely associated with pancreatic and biliary tract cancers [4]. Usually, the concentration of CA19-9 is significant less than 37 U/mL in normal healthy human blood [5]. A slight increase of CA19-9 implies the possibility of pancreatic cancer development [6]. Up to 63.2 U/mL and 92 U/mL indicate the possibility of cholangiocarcinoma and malignant pancreatic neoplasms, respectively [7,8]. Considering that CA19-9 has been identified as the most reliable biomarker for pancreatic and biliary tract cancers, highly sensitive and convenient detection of CA19-9 is urgent [9].

In recent years, various methods have been developed to detect CA19-9 including enzyme-linked immunosorbent assays (ELISA) [10], surface-enhanced Raman scattering spectroscopy (SERS) [11], radio-immunoassay (RIA) [12], photoelectrochemistry (PEC), [13] and chemiluminescence (CL) [14]. These methods usually require complex and tedious operations, expensive instruments and professional technicians. Electrochemical immunosensor has become one of the most widely analytical methods due to the advantage of low equipment cost, high detection sensitivity, simple instrument operation, fast response, and online or real-time monitoring [15,16,17]. Electrochemical immunosensor could fall into two categories, namely: free redox probes in solution [18,19] and immobilization of redox probes [20,21] onto the electrode surface. Compared with the former method, electrochemical immunosensors based on the immobilized redox probes have the advantages of reagentless detection and simple operations. Therefore, the exploitation of various materials to design a highly sensitive electrochemical immunosensing interface with convenient immobilization of redox probes and further biological recognitive species is highly desirable.

Carbon nanomaterials (e.g., graphene, carbon nanotubes, carbon quantum dots) as functional blocks (e.g., enhanced materials, electrocatalysts, carriers, or probes) have received great attention for construction of various chemo/biosensors with high sensitivity [22,23,24,25,26,27,28]. Especially, graphene and its derivatives with high conductivity, larger specific surface area, and mechanical stability could accelerate the electron transfer between electroactive species and electrodes, which have become important functional materials for the fabrication of high-performance electrochemical sensors [29,30,31,32,33,34]. Owing to the π-conjugated two-dimensional planar structures and the oxygen-containing groups, reduced graphene oxides could not only combine with other materials with synergistical effects including carbon nanotubes [35], polymers [36], metal nanoparticles [37,38], and nanoporous films [39,40,41,42,43], but also provide amounts of binding site for immobilization of biological recognitive molecules, remarkably promoting the analytical performance.

Indium tin oxide (ITO)-coated glasses have the characteristics of good electrical conductivity, inexpensiveness, optical transparency, easy miniaturization, and disposability, which have been widely used as electrode substrates for the construction of electrochemical/electroluminescent sensors [44]. In this work, we report a simple and sensitive label-free electrochemical immunosensor for quantitative determination of CA19-9 by using an ITO electrode modified with electrochemically reduced graphene oxide and carbon nanotubes (ErGO-CNT) nanocomposite, and further probes immobilization. ErGO-CNT nanocomposite could be electrodeposited onto the ITO surface by a simple and controllable electrochemical method, greatly improving the electronic conductivity. Arising from the carboxyl group of CNT and the π-conjugated structure of ErGO-CNT, electrochemical probe, methylene blue (MB), could be immobilized onto the ErGO-CNT via electrostatic and π-π effects. With the linkage of polydopamine (PDA), the anti-CA19-9 antibody is able to covalently bind to the MB/ErGO-CNT/ITO, and the immunosensor is achieved with a wide linear range and low limit of detection. Furthermore, the proposed immunosensor has been successful applied to the analysis of human serum.

## 2. Results and Discussion

### 2.1. Principle of CA19-9 Detection

**Figure 1:**
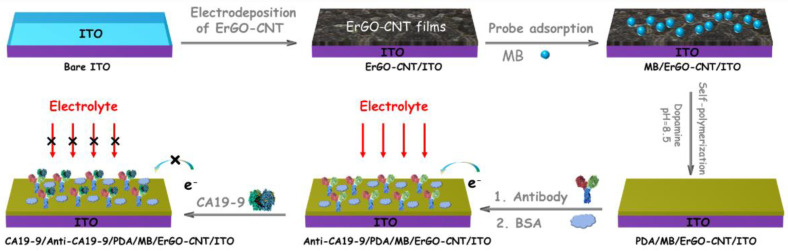
Schematic illustration for the label-free electrochemical determination of CA19-9.

Figure 1 illustrates the fabrication of an immunosensing interface and the reagentless determination of CA19-9 based on the electrochemical signals of the immobilized mediator. GO and CNT were pre-mixed to prepare the GO-CNT dispersion and underwent an electrochemical deposition process, leading to the reduction of GO and finally forming ErGO-CNT onto the ITO surface (ErGO-CNT/ITO). The hybrid of GO and CNT has two advantages. On the one hand, GO could enhance the solubility and dispersibility of CNT in an aqueous solution because of the π-π stacking interaction between the sidewalls of CNT and the aromatic region of GO. On the other hand, incorporated CNT serving as good electronic conductive wires is capable of promoting the electrochemical reduction of GO, decreasing the co-deposition potential. Such obtained ErGO-CNT nanocomposite could improve electroactive area and provide a binding site for cationic redox probe (e.g., MB). Due to the negative charge of carboxylated CNT and the π-conjugated structure of ErGO-CNT, MB with a positive charge can be adsorbed at the ErGO-CNT (MB/ErGO-CNT/ITO) through electrostatic and π-π interactions, giving rise to the electrochemical signals. Then, polydopamine (PDA) was formed on the MB/ErGO-CNT/ITO electrode surface through oxidative self-polymerization of dopamine (DA) in the alkaline solution, which could not only stabilize the inner layer of MB, but also act as the coupling agent for the immobilization of recognitive anti-CA19-9 antibody through Michael addition or Schiff-based reactions. After blocking the non-specific sites with bovine serum albumin (BSA), the immunosensor, denoted as Anti-CA19-9/PDA/MB/ErGO-CNT/ITO, was obtained. When CA19-9 specifically interacted with anti-CA19-9 on the surface of the electrode, the electrochemical signal was reduced due to the poor electron transport efficiency of proteins, realizing the reagentless and label-free electrochemical determination of CA19-9.

### 2.2. Characterization of GO-CNT Nanocomposite

**Figure 2:**
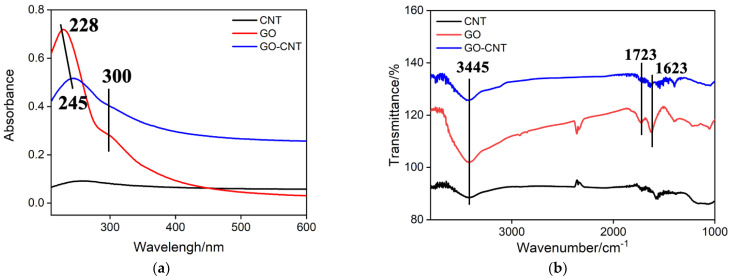
UV-Vis (**a**) and FTIR (**b**) spectra of the supernatant of CNT, GO, and GO-CNT nanocomposite.

Figure 2a shows the UV-Vis spectra of the supernatant of GO, CNT, and GO-CNT nanocomposite. As seen, CNT without dispersants has no obvious peaks, which arises from the precipitation of CNT after centrifugation. GO shows an obvious absorption peak at 228 nm and a shoulder peak at 300 nm, corresponding to the π→π* transitions of aromatic C-C bond and n→π* transition of C=O bond, respectively. For the GO-CNT nanocomposite, the absorption peak at 300 nm does not shift but that at 228 nm shifts bathochromically to 245 nm, which is due to the π-stacking interactions between the multiple aromatic regions of GO and the sidewalls of CNT [45]. FTIR was also employed to confirm the complex of GO and CNT. As shown in Figure 2b, a broad absorption band at 3445 cm^−1^ and two absorption bands at 1723 cm^−1^ and 1623 cm^−1^ are observed at the spectrum of GO, which is attributed to the hydroxyl, carbonyl, and carboxyl groups, respectively. By contrast, GO-CNT nanocomposite has similar absorption bands but not observed at the spectrum of CNT, suggesting the successful formation of GO-CNT nanocomposite. It is noteworthy that the presence of oxygen-containing groups in GO-CNT nanocomposite could provide abundant binding site for further immobilization of functional species.

### 2.3. Characterization of ErGO-CNT/ITO Electrode

**Figure 3:**
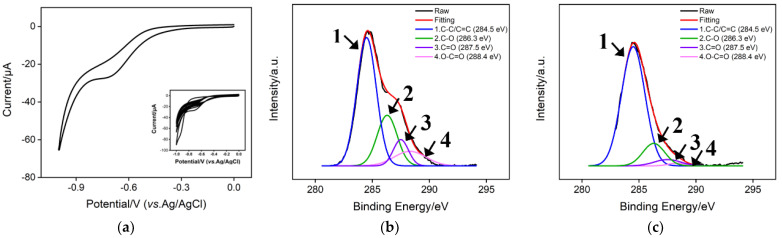
(**a**) CV curves of the bare ITO electrode in 0.2 M PBS (pH = 6.5) containing GO-CNT. Inset is the corresponding 30-successive CV curves. High-resolution C 1 s XPS spectra of (**b**) GO-CNT/ITO, (**c**) ErGO-CNT/ITO electrode.

**Figure 4:**
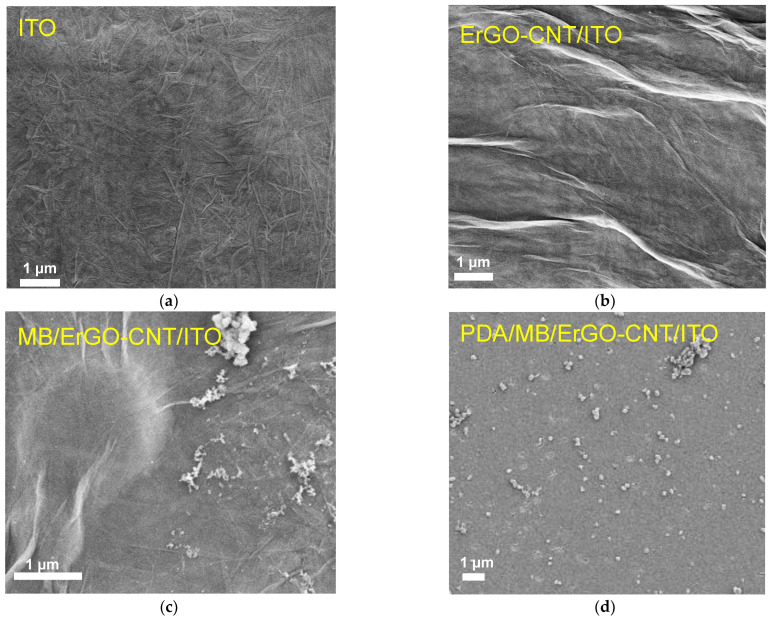
Top-view SEM images of bare ITO (**a**), ErGO-CNT/ITO (**b**), MB/ErGO-CNT/ITO (**c**), and PDA/MB/ErGO-CNT/ITO (**d**) electrodes.

Figure 3a shows the repetitive cyclic voltammetry (CV) curves of the bare ITO electrode in a potential range from 0 to −1.0 V. In this process, electrochemical reduction of GO occurs and cathodic peak current appears at −1.0 V, eventually resulting in the electrodeposition of ErGO-CNT onto the ITO surface. It is noteworthy that above cathodic peak current gradually decreased with the continuous scanning, further suggesting the reduction of GO and electrodeposition of ErGO-CNT. In order to confirm the electrochemical reduction of GO, the XPS technique was used. Figure 3b,c shows the XPS spectra of GO–CNT/ITO and ErGO–CNT/ITO electrodes. As seen, four characteristic peaks located at 284.5, 286.3, 287.5, and 288.4 eV are ascribed to the C-C/C=C, C-O, C=O, and O-C=O, respectively. When ErGO-CNT was deposited on the ITO electrode, the intensities of characteristic peaks related to oxygen-containing groups of carbon decreased, indicating that the electrodeposition of GO-CNTs was accompanied by the reduction of oxygen-containing functional groups. As shown in Figure 4a,b, numerous wrinkles of GO and bundled structures of CNT are observed, indicating the successful modification of ErGO–CNT on the ITO surface.

**Figure 5:**
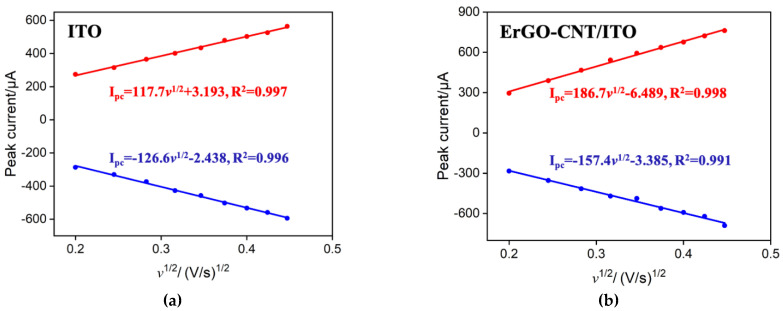
Relationship between peak current vs. square root of scan rate derived from the CV curves of bare ITO (**a**) and ErGO-CNT/ITO (**b**) electrodes obtained in 0.05 M KHP containing 0.5 mM K_3_[Fe(CN)_6_].

To examine the electrode performance of obtained ErGO-CNT/ITO, electrochemically active surface area (ECSA) of bare ITO and ErGO-CNT/ITO electrodes were compared. ECSA of bare ITO can be accurately calculated by using the Randles–Sevcik equation in the presence of reversible probe (K_3_[Fe(CN)_6_]) [46].

(1) $$I_{P}=2.69\times 10^{5}AD^{1/2}n^{3/2}v^{1/2}C$$

where *A*, *C*, *n*, *D*, and ν represent the effective surface area, the bulk concentration of K_3_[Fe(CN_6_)], the number of electrons transferred (*n* = 1), the diffusion coefficient of K_3_[Fe(CN_6_)] (6.67 × 10^−6^ cm^2^ s^−1^), and the scan rate, respectively. Figure 5 gives the linear relationship (*I*_p_-*ν*^1/2^) between the redox peak currents of 0.5 mM K_3_[Fe(CN)_6_] obtained from these two electrodes (bare ITO and ErGO-CNT/ITO) and the square root of scan rate. According to Equation 1, the ECSA of the bare ITO and ErGO-CNT/ITO were calculated to be 0.36 and 0.45 cm^2^. Despite a slight increase in ECSA, ErGO-CNT/ITO exhibits remarkable enrichment ability towards MB (Figure S1) due to the electrostatic attraction and π-π effect, showing the great potential for the construction of reagentless electrochemical sensors.

### 2.4. Stabilization Effect of PDA

**Figure 6:**
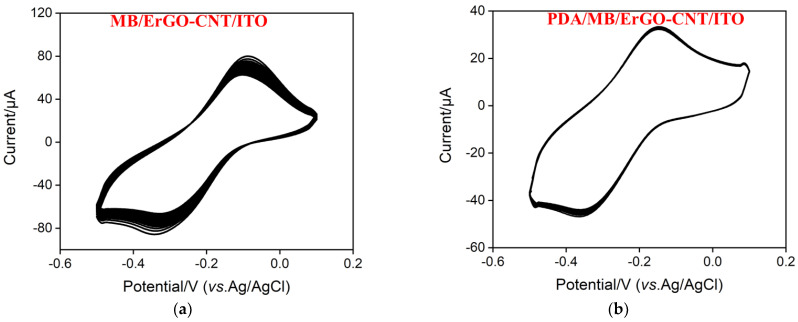
Successive CV curves of the (**a**) MB/ErGO-CNT/ITO and (**b**) PDA/MB/ErGO-CNT/ITO electrodes in 0.01 M PBS (pH = 7.4).

MB with a reversible redox and low-cost properties is an organic dye that belongs to the phenothiazine family, which has been widely used as an excellent electrochemical redox probe in the field of electrochemical sensors [47]. Cationic MB was immobilized onto the ErGO-CNT/ITO surface via electrostatic and π-π effects under stirring for 60 min (Figure S3), which did not change the surface morphology of ErGO-CNT/ITO (Figure 4b,c). After self-polymerization of PDA, smooth surface was presented at the PDA/MB/ErGO-CNT/ITO (Figure 4c,d). After self-polymerization of PDA, smooth surface was presented at the PDA/M), indicating the formation of uniform PDA film. Considering the protective effect of PDA in previous reports [48,49], the stability of PDA/MB/ErGO-CNT/ITO was investigated. Figure 6 compares the 20 consecutive CV scans of the prepared MB/ErGO-CNT/ITO electrode and PDA/MB/ErGO-CNT/ITO electrodes. As displayed, the MB/ErGO-CNT/ITO electrode has a pair of well-defined redox peaks, corresponding to the signal of MB and suggesting the successful immobilization of MB. However, the redox peak current signals of MB at the MB/ErGO-CNT/ITO decreased with an increase of scanning cycles while those at the PDA/MB/ErGO-CNT/ITO hardly changed. This is because the presence of PDA is able to prevent the inner MB from falling off the electrode surface and effectively stabilize the immobilization of MB. Moreover, the effect of pH of the supporting electrolyte on the PDA/MB/ErGO-CNT/ITO was studied. As shown in Figure S2, the anodic peak current increases with the decrease of pH value and the anodic current peak shifts positively, which is attributed to the proton-involved electrochemical reaction of MB [50,51]. However, considering the stable activity of the protein under neutral conditions and 91% of signal at the pH of 4.0, pH = 7.4 was chosen as the optimal experimental condition.

### 2.5. Fabrication of Immunosensor

**Figure 7:**
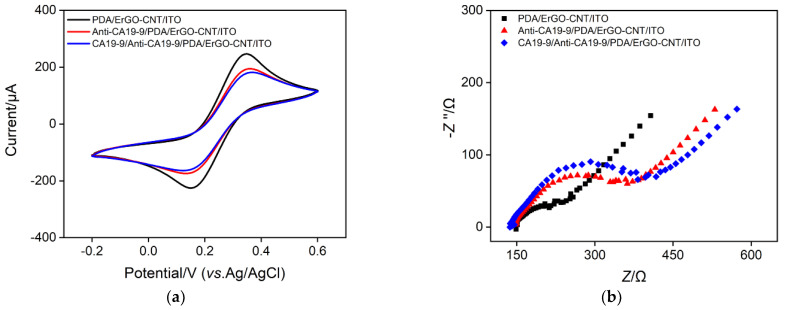

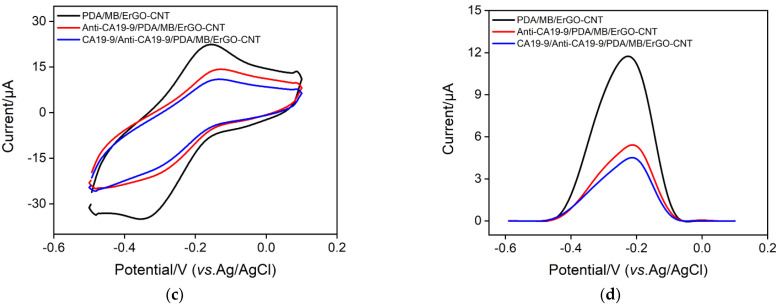
(**a**) CV and (**b**) EIS plots of different electrodes in 0.1 M KCl solution containing 2.5 mM Fe(CN)_6_^3–/4–^. (**c**) CV and (**d**) DPV curves of different electrodes in 0.01 M PBS (pH = 7.4).

The feasibility of the immunosensor construction was verified by the electrochemical method. Figure 7a is the CV curves of each electrode in 0.1 M KCl solution containing 2.5 mM Fe(CN)_6_^3–/4–^. As shown, Fe(CN)_6_^3–/4–^ displays a pair of reversible redox peaks at the PDA/MB/ErGO-CNT/ITO electrode. Then, recognitive antibody (anti-CA19-9) can be immobilized onto the PDA/MB/ErGO-CNT/ITO electrode surface based on the interaction between the dopaquinone structure of PDA and the –SH or –NH_2_ groups of anti-CA19-9. After blocking the non-specific sites with BSA, CA19-9 was detected by Anti-CA19-9/PDA/MB/ErGO-CNT/ITO electrode. Owning to the non-conductive steric hindrance layer of proteins, both anti-CA19-9 and CA19-9 hinder the electron exchange of electrochemical probes on the electrode surface, leading to the decreased redox peak signals and larger peak-to-peak difference. Figure 7b shows the EIS responses of each electrode to 2.5 mM Fe(CN)_6_^3–/4–^. Each curve consists of a semicircle in the high frequency region and a linear part in the low frequency region, representing an electron transfer-limited process and a diffusion-limited process, respectively. As seen, the charge transfer resistance was increased after each modification, which was consistent with the current variation of CV responses shown in Figure 7a. At the same time, we used the inner probe strategy to characterize the electrode construction process. It can be seen from Figure 7c,d, the CV and differential pulse voltammetry (DPV) signals of MB gradually decreased with the stepwise modification. All above results demonstrate the successful construction of the immunosensor.

### 2.6. Electrochemical Determination of CA19-9

**Figure 8:**
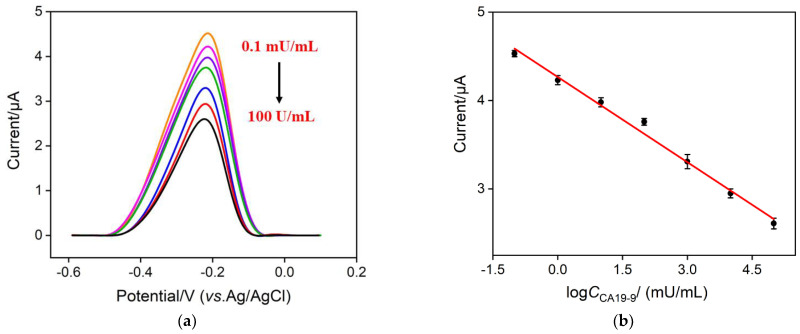
(**a**) DPV response of the immunosensor to different concentrations of CA19-9, from 0.1 mU/mL to 100 U/mL. (**b**) Calibration curve of the proposed electrochemical immunosensor. Error bars represent the standard deviations of three measurements.

CA19-9 was detected by DPV based on the decrease of the electrochemical signal of MB after CA19-9 binding, and the results were shown in Figure 8. As shown, after incubating with different concentrations of CA19-9, anodic peak currents decrease with the increasing CA19-9 concentration. This is because the formed antigen–antibody complex hinders the access of electrolyte anions to the electrode surface and charge compensation could not occur. The good linear relationship between anodic peak currents and the logarithm of CA19-9 concentration could be found from 0.1 mU/mL to 100 U/mL (Figure 8b), yielding a regressive equation of *I*_EC_ = –0.321 lg*C*_CA19-9_ + 4.27 (*R*^2^ = 0.990) and the limit of detection (LOD) of 0.54 nU/mL (S/N = 3). Analytical performance of our sensor with some other reported methods is provided in Table 1. As compared, our proposed sensor has a wider linear range and a rather lower LOD.

### 2.7. Selectivity, Reproducibility, and Stability of the Constructed Immunosensor

**Figure 9:**
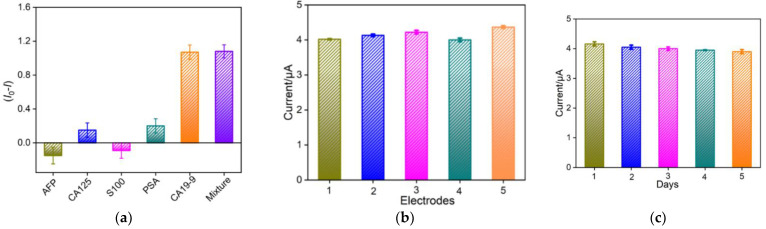
(**a**) Selectivity of the electrochemical immunosensors to CA19-9. (1) 1 ng/mL AFP; (2) 1 U/mL CA125; (3) 1 ng/mL S100; (4) 1 ng/mL PSA; (5) 100 mU/mL CA19-9. Reproducibility (**b**,**c**) stability of immunosensor. The concentration of CA19-9 is 1 mU/mL. Error bars represent the standard deviations of three measurements.

The selectivity of the constructed immunosensor was investigated. As shown in Figure 9a, except for CA19-9, the other four antigens including AFP, CA125, S100, and PSA did not change the peak current responses at the anti-CA19-9/PDA/MB/ErGO-CNT/ITO sensor. Even though all of tumor markers were mixed with CA19-9, the peak current of the electrode was nearly similar with that of a single CA19-9, proving the good selectivity of the developed immunosensor. The inter-electrode reproductivity and storage stability of the constructed immunosensor were also evaluated. The reproducibility of this immunosensor was investigated by preparing five electrodes in the same batch and the obtained RSD value of five different electrodes was 3.6% (Figure 9b). After 5 days of storage in a refrigerator (4 ℃), the response of the immunosensor towards CA19-9 retained about 94% of the initial signal (Figure 9c). All these results show that the proposed immunosensor has good selectivity, reproducibility, and stability, making a good candidate for real sample analysis.

### 2.8. Determination of CA19-9 in Human Serum

In order to evaluate the practical application potential of our immunosensor, 50-time diluted human serum with 0.01 M PBS (pH = 7.4) was employed as the analytical samples. Table 2 shows the results of human serum samples with artificially spiking in a known concentration of CA19-9, exhibiting satisfactory recoveries (between 95.0 and 110%) and small RSDs (between 0.36 and 1.3%, *n* = 3). Moreover, the human serum sample spiked with 10 U/mL was determined by our sensor and enzyme-linked immuno-sorbent assay (ELISA) and the obtained results of both methods were comparable, showing the reliability and accuracy of our immunosensor.

## 3. Materials and Methods

### 3.1. Chemicals and Materials

Carbohydrate antigen 19-9 (CA19-9), carcinoembryonic antigen (CEA), prostate specific antigen (PSA) and carcinoma antigen 125 (CA125), and CA19-9 antibody (anti-CA19-9) were purchased from Beijing KEY-BIO Biotech Co., Ltd. (China). S100 calcium binding protein β (S100) was ordered from Proteintech (Wuhan, China) and alpha-fetoprotein (AFP) was bought from Nanjing OkayBio (China). NaH_2_PO_4_, Na_2_HPO_4_, KCl, and dopamine (DA) were purchased from Aladdin Chemistry (Shanghai, China). Bovine serum albumin (BSA) was obtained from Macklin (Shanghai, China). Multi-walled carbon nanotubes (CNT, OD < 8 nm, length ~ 30 μm, 95%) were bought from Chengdu Institute of Organic Chemistry, Chinese Academy of Sciences. Methylene blue trihydrate (MB) was purchased from Tianjin Yongda Chemical Reagent Co. Ltd. (China). Human serum was provided by Shanxi Bethune Hospital (Taiyuan, China). ITO-coated glasses (<17 Ω/square, thickness: 100 ± 20 nm) were obtained from Zhuhai Kaivo Optoelectronic Technology. To remove impurities on the ITO surface, the electrode was first immersed in 1 M NaOH aqueous solution overnight and then sonicated in acetone, ethanol, and deionized water for 30 min, respectively. All aqueous solutions throughout this work were prepared in ultrapure water (18.2 MΩ cm).

### 3.2. Experiments and Instrumentations

Scanning electron microscopy (SEM) was conducted on a field-emission scanning electron microscope (S-4800, Hitachi, Tokyo, Japan). Cyclic voltammetry (CV) and differential pulse voltammetry (DPV) measurements were performed on an Autolab PGSTAT302N electrochemical workstation (Metrohm, Herisau, Switzerland). Above electrochemical measurements were performed with a three-electrode system containing a bare or modified ITO as the working electrode, Ag/AgCl (saturated KCl solution) as the reference electrode, and a Pt wire electrode as the counter electrode. DPV measurement parameters include step (0.005 V), modulation amplitude (0.05 V), modulation time (0.05 s), and interval time (0.2 s). A PHI5300 electron spectrometer (PE Ltd., Waltham, MA., USA) was used to conduct X-Ray photoelectron spectroscopy (XPS) analysis (250 W, 14 kV, Mg Kα radiation). Ultraviolet-Vis (UV-Vis) absorption spectra were obtained from a UV-Vis spectrometer (UV-2450; Shimadzu, Kyoto, Japan). Fourier transform infrared spectroscopy (FTIR) data were recorded on a Vertex 70 spectrometer (Bruker, Woodlands, TX., USA) through KBr tablet method.

### 3.3. Preparation of the MB/ErGO-CNT/ITO

GO was prepared by a modified Hummers method [55]. The fabrication process of GO-CNT dispersion is as the following: 1 mg/mL GO and 0.5 mg/mL CNT was mixed into PBS (0.2 M pH = 6.5) solution and sonicated for 1 h. The unstable CNT was removed by centrifugation at 8000 rpm for 10 min. To deposit the ErGO-CNT on the ITO, the clean ITO electrode (geometric area = 0.5 cm^2^) was soaked into the above GO-CNT dispersion and applied 30 successive CVs scanning from 0 to –1.0 V at the scan rate of 50 mV/s. In this process, electrochemical reduction of GO and electrodeposition of their nanocomposite to the ITO electrode surface simultaneously occur. The obtained electrode was rinsed with water lightly, dried at 60 °C and applied a constant current of –350 mA for 10 s in 0.2 M PBS (pH = 6.5) to further reduce GO. ErGO-CNT supported by ITO electrode was finally prepared, termed as ErGO-CNT/ITO. MB/ErGO-CNT/ITO was obtained by immersing the ErGO-CNT/ITO electrode into a 0.01 M PBS (pH = 7.4) solution containing 1 mM MB under stirring for 60 min.

### 3.4. Fabrication of Immunosensor

In order to immobilize antibodies and encapsulate MB, the MB/ErGO-CNT/ITO electrode was first immersed in DA solution (1 mg/mL in 0.1 M PBS, pH = 8.5) for 1 h and self-polymerization of the DA was induced to obtain the polydopamine (PDA)/MB/ErGO-CNT/ITO electrode. After removing the residual DA, 50 μL of 100 μg/mL anti-CA19-9 in 0.01 M PBS (pH 7.4) was dropped on the above electrode surface and incubated at 4 °C overnight. Loosely bounded anti-CA19-9 antibody was carefully washed off and then 50 μL of 1 wt% BSA in 0.01 M PBS (pH 7.4) was used to block nonspecific binding sites at 37◦C for 1 h. Finally, 50 μL of various concentrations of CA19-9 was dropped onto the immunosensor surface and incubated at 37◦C for 0.5 h. After being rinsed with PBS, the DPV curves were recorded to realize the quantitative detection of CA19-9.

## 4. Conclusions

In this work, a simple and sensitive label-free electrochemical immunosensor is fabricated for reagentless determination of CA19-9 through ErGO-CNT nanocomposite and surface-immobilized MB probe. ErGO-CNT could be prepared by a simple and controllable electrodeposited method. The carboxyl group of CNT and the π-conjugated structure of ErGO-CNT provide binding sites for the cationic MB probe, which could be further stabilized by PDA. With the linkage of PDA, anti-CA19-9 antibody covalently binding to the MB/ErGO-CNT/ITO endows the electrode recognized capacity. Combining with the good conductivity of ErGO-CNT and stabilization of PDA, determination of CA19-9 was achieved by recording the reduction of redox signals of MB after incubation with CA19-9. Furthermore, the fabricated immunosensor exhibits convenient construction steps, high sensitivity, good selectivity, and has been successfully applied to the analysis in human serum, providing an easy and efficient strategy for sensitive determination of CA19-9.

## Figures and Tables

**Table 1:** Comparison of detection performance of our sensor with some other reported methods for the determination of CA19-9.

Method	Electrode	Linear Range (mU/mL)	LOD (mU/mL)	Refs.
ECL	GO/HBP/ITO	2–5 × 10^4^	0.25	[52]
ECL	MWCNT–Pt– Luminol-GCE	0.1–1 × 10^4^	0.046	[53]
EIS	CeO_2_/FeOx@mC500/AuE	0.1–1 × 10^4^	0.01	[6]
DPV	Au/GO-MA/GCE	0.1–1 × 10^5^	0.032	[54]
DPV	PDA/MB/GO-CNT/ITO	0.1–1 × 10^5^	0.00054	This work

GO/HBP: GO-grafted hyperbranched/aromaticpolyamide; MWCNT–Pt–Luminol: multi-walled carbon nanotube/platinum/luminol nanocomposites; CeO_2_/FeOx@mC500: bimetallic cerium and ferric oxide nanoparticles embedded within the mesoporous carbon matrix; Au/GO-MA: Au graphene oxide-melamine.

**Table 2:** Determination of CA19-9 in real samples ^a^.

Sample	Added (mU/mL)	Found (mU/mL)	Recovery (%)	RSD (%)
Serum ^a^	0.100	0.0950	95.0	0.36
	1.00	1.10	110	0.50
	1000	952	95.2	1.3

^a^: 50-time diluted with PBS (0.01 M, pH = 7.4).

## Data Availability

The data presented in this study are available on request from the corresponding author.

## Supplementary Materials

The following supporting information can be downloaded at: https://www.mdpi.com/article/10.3390/molecules27206778/s1, Figure S1: CV curves of bare ITO and ErGO-CNT/ITO electrodes after soaking into MB solution; Figure S2: Effect of pH of supporting electrolyte on the PDA/MB/GO-CNT/ITO electrode; Figure S3: Optimization of accumulation time of MB.

## Sample Availability

Not available.

## References

[1] Wu Y., Tilley R., Gooding J. (2018). Challenges and solutions in developing ultrasensitive biosensors. J. Am. Chem. Soc..

[2] Zheng Y., Zhao L., Ma Z. (2018). pH responsive label-assisted click chemistry triggered sensitivity amplification for ultrasensitive electrochemical detection of carbohydrate antigen 24-2. Biosens. Bioelectron..

[3] Zhao Y., Cui L., Sun Y., Zheng F., Ke W. (2018). Ag/CdO NP-engineered magnetic electrochemical aptasensor for prostatic specific antigen detection. ACS Appl. Mater. Interfaces.

[4] Lodhi F., Ali M., Hussain R., Iftikhar (2006). Serum tumor markers. Prof. Med. J. Mar..

[5] Imaoka H., Shimizu Y., Senda Y., Natsume S., Mizuno N., Hara K., Hijioka S., Hieda N., Tajika M., Tanaka T. (2016). Post-adjuvant chemotherapy CA19-9 levels predict prognosis in patients with pancreatic ductal adenocarcinoma: A retrospective cohort study. Pancreatology.

[6] Wang M., Hu M., Hu B., Guo C., Song Y., Jia Q., He L., Zhang Z., Fang S. (2019). Bimetallic cerium and ferric oxides nanoparticles embedded within mesoporous carbon matrix: Electrochemical immunosensor for sensitive detection of carbohydrate antigen 19-9. Biosens. Bioelectron..

[7] Bhattarai A., Jha B., Timilsina S. (2015). Serum CA19-9 levels in benign and malignant diseases associated with the gastrointestinal tract. ACCLM.

[8] Duraker N., Hot S., Polat Y., Hobek A., Gencler N., Urhan N. (2007). CEA, CA19-9, and CA125 in the differential diagnosis of benign and malignant pancreatic diseases with or without jaundice. J. Surg. Oncol..

[9] Li W., Shu D., Zhang D., Ma Z. (2018). Multi-amplification of the signal of voltammetric immunosensors: Highly sensitive detection of tumor marker. Sens. Actuators B Chem..

[10] Wang W., Xu X., Tian B., Wang Y., Du L., Sun T., Shi Y., Zhao X., Jing J. (2017). The diagnostic value of serum tumor markers CEA, CA19-9, CA125, CA15-3, and TPS in metastatic breast cancer. Clin. Chim. Acta.

[11] Tian Y., Li X., Wang F., Gu C., Zhao Z., Si H., Jiang T. (2021). SERS-based immunoassay and degradation of CA19-9 mediated by gold nanowires anchored magnetic–semiconductor nanocomposites. J. Hazard. Mater..

[12] Terenghi M., Elviri L., Careri M., Mangia A., Lobinski R. (2009). Multiplexed determination of protein biomarkers using metal-tagged antibodies and size exclusion chromatography−inductively coupled plasma mass spectrometry. Anal. Chem..

[13] Zhu H., Fan G., Abdel-Halim E.S., Zhang J., Zhu J. (2016). Ultrasensitive photoelectrochemical immunoassay for CA19-9 detection based on CdSe@ZnS quantum dots sensitized TiO_2_ NWs/Au hybrid structure amplified by quenching effect of Ab_2_@V^2+^ conjugates. Biosens. Bioelectron..

[14] Shi M., Zhao S., Huang Y., Zhao L., Liu Y. (2014). Signal amplification in capillary electrophoresis based chemiluminescent immunoassays by using an antibody-gold nanoparticle-DNAzyme assembly. Talanta.

[15] Gong J., Zhang T., Luo T., Luo X., Yan F., Tang W., Liu J. (2022). Bipolar silica nanochannel array confined electrochemiluminescence for ultrasensitive detection of SARS-CoV-2 antibody. Biosens. Bioelectron..

[16] Gong J., Zhang T., Chen P., Yan F., Liu J. (2022). Bipolar silica nanochannel array for dual-mode electrochemiluminescence and electrochemical immunosensing platform. Sens. Actuators B Chem..

[17] Thangapandi K., Arumugam S., Amalesh N., Ajeet K., Saikat K. (2021). Bio-nanocomposite based highly sensitive and label-free electrochemical immunosensor for endometriosis diagnosticsapplication. Bioelectrochemistry.

[18] Chang Q., Huang J., Xi F. (2022). Simple immunosensor for ultrasensitive electrochemical determination of biomarker of bone metabolism in human serum. Front. Chem..

[19] Ma K., Zheng Y., An L., Liu J. (2022). Ultrasensitive immunosensor for prostate-specific antigen based on enhanced electrochemiluminescence by vertically ordered mesoporous silica-nanochannel film. Front. Chem..

[20] Zhang J., Yang L., Pei J., Tian Y., Liu J. (2022). A reagentless electrochemical immunosensor for sensitive detection of carcinoembryonic antigen based on the interface with redox probe-modified electron transfer wires and effectively immobilized antibody. Front. Chem..

[21] Lin J., Li K., Wang M., Chen X., Liu J., Tang H. (2020). Reagentless and sensitive determination of carcinoembryonic antigen based on a stable Prussian blue modified electrode. RSC Adv..

[22] Wei X., Luo X., Xu S., Xi F., Zhao T. (2022). A flexible electrochemiluminescence sensor equipped with vertically ordered mesoporous silica nanochannel film for sensitive detection of clindamycin. Front. Chem..

[23] Xuan L., Liao W., Wang M., Zhou H., Ding Y., Yan F., Liu J., Tang H., Xi F. (2021). Integration of vertically-ordered mesoporous silica-nanochannel film with electro-activated glassy carbon electrode for improved electroanalysis in complex samples. Talanta.

[24] Yan L., Zhang C., Xi F. (2022). Disposable amperometric label-free immunosensor on chitosan-graphene-modified patterned ito electrodes for prostate specific antigen. Molecules.

[25] Zheng W., Su R., Lin X., Liu J. (2022). Nanochannel array modified three-dimensional graphene electrode for sensitive electrochemical detection of 2,4,6-trichlorophenol and prochloraz. Front. Chem..

[26] Deng X., Zhao J., Ding Y., Tang H., Xi F. (2021). Iron and nitrogen co-doped graphene quantum dots as highly active peroxidases for the sensitive detection of l-cysteine. New J. Chem..

[27] Xi F., Zhao J., Shen C., He J., Chen J., Yan Y., Li K., Liu J., Chen P. (2019). Amphiphilic graphene quantum dots as a new class of surfactants. Carbon.

[28] Zhao J., Zheng Y., Pang Y., Chen J., Zhang Z., Xi F., Chen P. (2020). Graphene quantum dots as full-color and stimulus responsive fluorescence ink for information encryption. J. Colloid Interface Sci..

[29] Sun D., Li H., Li M., Li C., Qian L., Yang B. (2019). Electrochemical immunosensors with AuPt-vertical graphene/glassy carbon electrode for alpha-fetoprotein detection based on label-free and sandwich-type strategies. Biosens. Bioelectron..

[30] Gong J., Tang H., Wang M., Lin X., Wang K., Liu J. (2022). Novel three-dimensional graphene nanomesh prepared by facile electro-etching for improved electroanalytical performance for small biomolecules. Mater. Des..

[31] Zhu X., Xuan L., Gong J., Liu J., Wang X., Xi F., Chen J. (2022). Three-dimensional macroscopic graphene supported vertically-ordered mesoporous silica-nanochannel film for direct and ultrasensitive detection of uric acid in serum. Talanta.

[32] Lu L., Zhou L., Chen J., Yan F., Liu J., Dong X., Xi F., Chen P. (2018). Nanochannel-confined graphene quantum dots for ultrasensitive electrochemical analysis of complex samples. ACS Nano.

[33] Zhou H., Dong G., Sailjoi A., Liu J. (2022). Facile pretreatment of three-dimensional graphene through electrochemical polarization for improved electrocatalytic performance and simultaneous electrochemical detection of catechol and hydroquinone. Nanomaterials.

[34] Su R., Tang H., Xi F. (2022). Sensitive electrochemical detection of p-nitrophenol by pre-activated glassy carbon electrode integrated with silica nanochannel array film. Front. Chem..

[35] Zhou H., Ma X., Sailjoi A., Zou Y., Lin X., Yan F., Su B., Liu J. (2022). Vertical silica nanochannels supported by nanocarbon composite for simultaneous detection of serotonin and melatonin in biological fluids. Sens. Actuators B Chem..

[36] Li J., Liu S., Yu J., Lian W., Cui M., Xu W., Huang J. (2013). Electrochemical immunosensor based on graphene-polyaniline composites and carboxylated graphene oxide for estradiol detection. Sens. Actuators B Chem..

[37] Wang A., You X., Liu H., Zhou J., Chen Y., Zhang C., Ma K., Liu Y., Ding P., Qi Y. (2022). Development of a label free electrochemical sensor based on a sensitive monoclonal antibody for the detection of tiamulin. Food Chem..

[38] Liu Q., Zhong H., Chen M., Zhao C., Liu Y., Xi F., Luo T. (2020). Functional nanostructure-loaded three-dimensional graphene foam as a non-enzymatic electrochemical sensor for reagentless glucose detection. RSC Adv..

[39] Yan F., Luo T., Jin Q., Zhou H., Sailjoi A., Dong G., Liu J., Tang W. (2021). Tailoring molecular permeability of vertically-ordered mesoporous silica-nanochannel films on graphene for selectively enhanced determination of dihydroxybenzene isomers in environmental water samples. J. Hazard. Mater..

[40] Yan F., Chen J., Jin Q., Zhou H., Sailjoi A., Liu J., Tang W. (2020). Fast one-step fabrication of a vertically-ordered mesoporous silica-nanochannel film on graphene for direct and sensitive detection of doxorubicin in human whole blood. J. Mater. Chem. C.

[41] Zhou H., Ding Y., Su R., Lu D., Tang H., Xi F. (2022). Silica nanochannel array film supported by ß-cyclodextrin-functionalized graphene modified gold film electrode for sensitive and direct electroanalysis of acetaminophen. Front. Chem..

[42] Ma K., Yang L., Liu J., Liu J. (2022). Electrochemical sensor nanoarchitectonics for sensitive detection of uric acid in human whole blood based on screen-printed carbon electrode equipped with vertically-ordered mesoporous silica-nanochannel film. Nanomaterials.

[43] Xi F., Xuan L., Lu L., Huang J., Yan F., Liu J., Dong X., Chen P. (2019). Improved adhesion and performance of vertically-aligned mesoporous silica-nanochannel film on reduced graphene oxide for direct electrochemical analysis of human serum. Sens. Actuators B Chem..

[44] Aydın E., Sezgintürk M. (2017). Indium tin oxide (ITO): A promising material in biosensing technology. TrAC Trends Anal. Chem..

[45] Zhang C., Ren L., Wang X., Liu T. (2010). Graphene oxide-assisted dispersion of pristine multiwalled carbon nanotubes in aqueous media. J. Phys. Chem. C.

[46] Alam A., Deen M. (2020). Bisphenol A electrochemical sensor using graphene oxide and beta-cyclodextrin-functionalized multi-walled carbon nanotubes. Anal. Chem..

[47] Lin C., Wu Y., Luo F., Chen D., Chen X. (2014). A label-free electrochemical DNA sensor using methylene blue as redox indicator based on an exonuclease III-aided target recycling strategy. Biosens. Bioelectron..

[48] Liu Y., Ai K., Lu L. (2014). Polydopamine and its derivative materials: Synthesis and promising applications in energy, environmental, and biomedical fields. Chem. Rev..

[49] Liu J., Wang J., Wang T., Li D., Xi F., Wang J., Wang E. (2015). Three-dimensional electrochemical immunosensor for sensitive detection of carcinoembryonic antigen based on monolithic and macroporous graphene foam. Biosens. Bioelectron..

[50] He Y., Ding L., Su B. (2015). Vertically ordered silica mesochannels as preconcentration materials for the electrochemical detection of methylene blue. Sci. China Chem..

[51] Yang S., Liu D., Meng Q., Wu S., Song X. (2018). Reduced graphene oxide-supported methylene blue nanocomposite as a glucose oxidase-mimetic for electrochemical glucose sensing. RSC Adv..

[52] Bahari D., Babamiri B., Salimi A., Hallaj R., Amininasab S. (2020). A self-enhanced ECL-RET immunosensor for the detection of CA19-9 antigen based on Ru(bpy)_2_(phen-NH_2_)(2+)-amine-rich nitrogen-doped carbon nanodots as probe and graphene oxide grafted hyperbranched aromatic polyamide as platform. Anal. Chim. Acta.

[53] Zhang X., Ke H., Wang Z., Guo W., Zhang A., Huang C., Jia N. (2017). An ultrasensitive multi-walled carbon nanotube-platinum-luminol nanocomposite-based electrochemiluminescence immunosensor. Analyst.

[54] Zhang N., Zhang D., Chu C., Ma Z. (2020). Label-assisted chemical adsorption triggered conversion of electroactivity of sensing interface to achieve the Ag/AgCl process for ultrasensitive detection of CA 19-9. Anal. Chim. Acta.

[55] Santhiago M., Maroneze C., Silva C., Camargo M., Kubota L. (2015). Electrochemical oxidation of glassy carbon provides similar electrochemical response as graphene oxide prepared by tour or hummers routes. ChemElectroChem.

