# Supplementary material for: Probe-Integrated Label-Free Electrochemical Immunosensor Based on Binary Nanocarbon Composites for Detection of CA19-9
Source: Molecules. 2022 Oct 11;27(20):6778. doi: 10.3390/molecules27206778 (PMC9607002; doi:10.3390/molecules27206778)
Supplement: Supplementary file 1 [file molecules-27-06778-s001.zip › molecules-1900635-supplementary.pdf]

Supporting Information to

Probe-Integrated Label-Free Electrochemical Immunosensor Based on Binary Nanocarbon Composites for Detection of CA19-9

Zhengzheng Yan [1], Jun Xing [2,*], Ruochong He [1], Qinping Guo [1] and Ji Li [1]

[1] General Surgery Department, Shanxi Bethune Hospital, Shanxi Academy of Medical Sciences, Tongji Shanxi Hospital, Third Hospital of Shanxi Medical University, Taiyuan 030032, China

[2] Department of Breast Surgery, Shanxi Bethune Hospital, Shanxi Academy of Medical Sciences, Tongji Shanxi Hospital, Third Hospital of Shanxi Medical University, Taiyuan 030032, China

* Correspondence: xingjun2022@sxmu.edu.cn

Table of Content

S1. CV curves of bare ITO and ErGO-CNT/ITO electrodes after soaking into MB solution

S2. Effect of pH of supporting electrolyte on the PDA/MB/GO-CNT/ITO electrode

S3. Optimization of accumulation time of MB

S1. CV curves of bare ITO and ErGO-CNT/ITO electrodes after soaking into MB solution

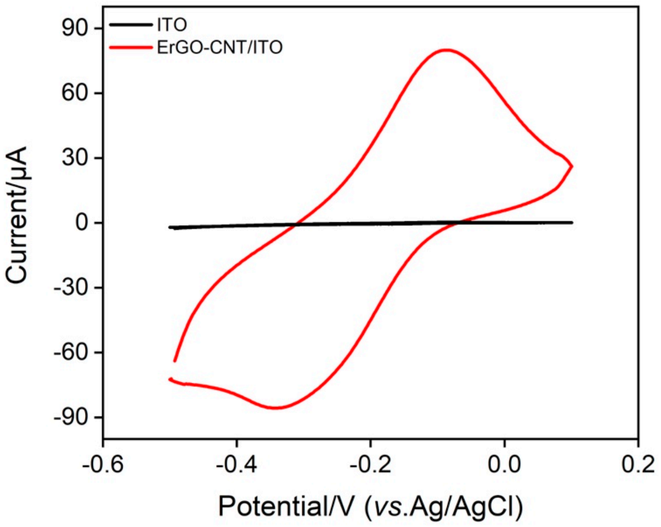

Figure S1. CV curves of bare ITO and ErGO-CNT/ITO electrodes in 0.01 M PBS (pH = 7.4) after soaking into 1 mM MB under stirring for 60 min.

S2 Effect of pH of supporting electrolyte on the PDA/MB/GO-CNT/ITO electrode

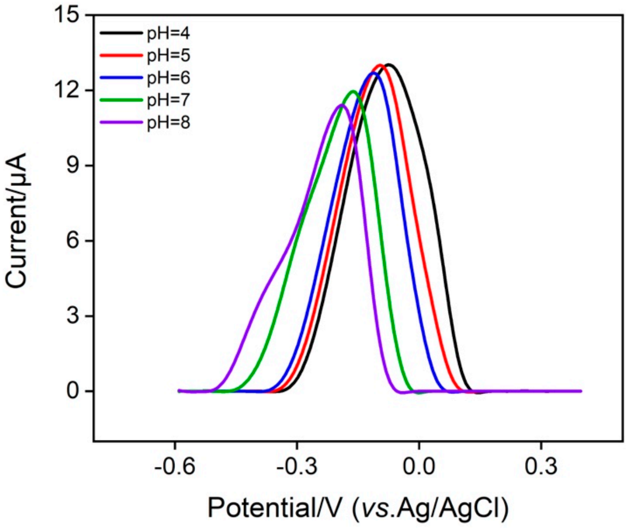

Figure S2. DPV curves of PDA/MB/GO-CNT/ITO electrode in 0.01 M PBS with different pH.

S3 Optimization of accumulation time of MB

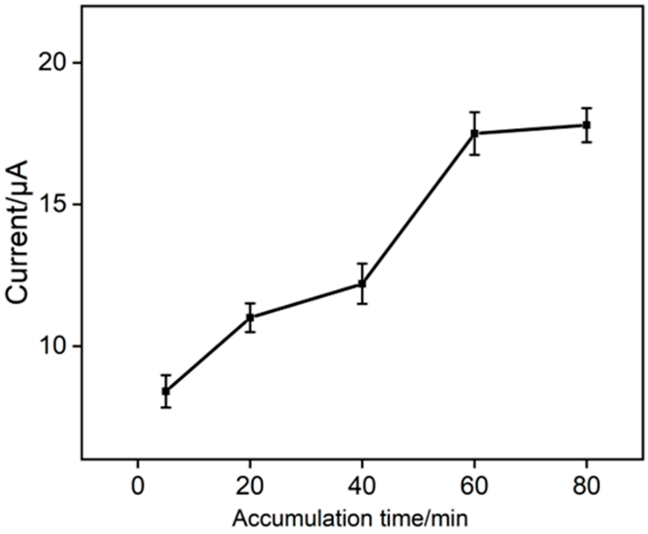

Figure S3. Effect of accumulation time of MB on current response of MB/ErGO-CNT/ITO electrode.
